# Development of a workflow for the detection of vancomycin-resistant *Enterococcus faecium* and *Enterococcus faecalis* from rectal swabs using the spectra VRE medium

**DOI:** 10.1186/s12941-023-00552-8

**Published:** 2023-01-06

**Authors:** Kendall Kling, Javier Rios, Laura Dirnberger, Wanda Polanco, Kevin Fritz, Michael Malczynski, Teresa Zembower, Chao Qi

**Affiliations:** 1 Clinical Microbiology Laboratory, Department of Pathology, Northwestern University Feinberg School of Medicine, 710 N Fairbanks Ct, Chicago, IL 60611 USA; 2 Department of Medicine, Division of Infectious Diseases, Northwestern University Feinberg School of Medicine, 676 N. St. Clair Street Suite 940, Chicago, IL 60611 USA

**Keywords:** Vancomycin-resistant enterococci, VRE, Spectra™ VRE agar, Infection prevention

## Abstract

**Background:**

Spectra™ VRE agar (Remel, Lenexa, KS) is a chromogenic agar that is FDA approved for screening patients for VRE colonization. The package insert recommends confirming isolates with identification and susceptibility testing, but confirming every culture delays time to result. Given the agar’s historic high specificity for *E. faecium* isolates, we theorized the agar could be utilized as a stand-alone screening to minimize reagents and time.

**Aim:**

Our laboratory sought to develop a workflow to optimize the use of the medium.

**Methods:**

We plated 3,815 rectal swabs to the Spectra VRE agar and compared results to traditional identification and susceptibility testing.

**Results:**

Dark blue or purple colonies on the agar demonstrated a sensitivity of 98% and specificity of 85% for detection of VRE *faecium*, but light blue colonies were significantly less specific for *E. faecalis*.

**Conclusions:**

We streamlined our workflow to accept dark blue or purple colonies as VRE *faecium* and plan to perform additional testing only on light blue colonies. Interestingly, higher quantity of growth increased the accuracy of the agar. In the future, growth quantity may be used to further streamline the workflow once more data is obtained.

## Introduction

Vancomycin-resistant enterococci (VRE), first described in 1988 in England, constitute a group of multi-drug-resistant organisms (MDROs) that are of major importance to healthcare epidemiology due to their ability to be hospital acquired [1]. Enterococci constitute the second most common cause of all central line-associated blood stream infections, the second most common cause of catheter-associated urinary tract infections, and the fourth most common cause of healthcare-associated skin and soft tissue infection [2, 3]. The National Nosocomial Infection Surveillance (NNIS) System of the CDC reported a 20% increase in nosocomial VRE from 1989 to 1993 [4]. Enterococci are able to persist in hospital environments due to their ability to colonize the GI tract, ability to exchange genetic information, and their relatively high intrinsic antibiotic resistance [2]. A meta-analysis in 2015 found an odds ratio of 2.5 for mortality risk for VRE versus vancomycin-susceptible enterococci (VSE) bacteremia [5].

In an attempt to curb this nosocomial enterococcal surge, infection prevention professionals recommended practices such as screening and isolation precautions for patients at risk for or colonized with VRE. A systematic review found that VRE colonization greatly increased risk for invasive infection, particularly in patients with malignancy [6]. The methodology that has become the gold standard for screening is the rectal swab culture [7]. In 2007, the impact of VRE surveillance cultures was evaluated in a retrospective cohort study that found that screening led to a 2.2-17-fold increase in VRE detection and enabled clinicians to initiate contact precautions 11 days earlier compared with use of clinical cultures alone [8]. The traditional culture methodology, however, has drawbacks as it is time consuming and requires multiple steps including growth from solid media, organism identification, and in-vitro susceptibility testing. Two alternative VRE screening methods emerged to avoid these delays, namely polymerase chain reaction (PCR) assays targeting *vanA* and/or *vanB* gene(s) and chromogenic agar.

**Fig. 1:**
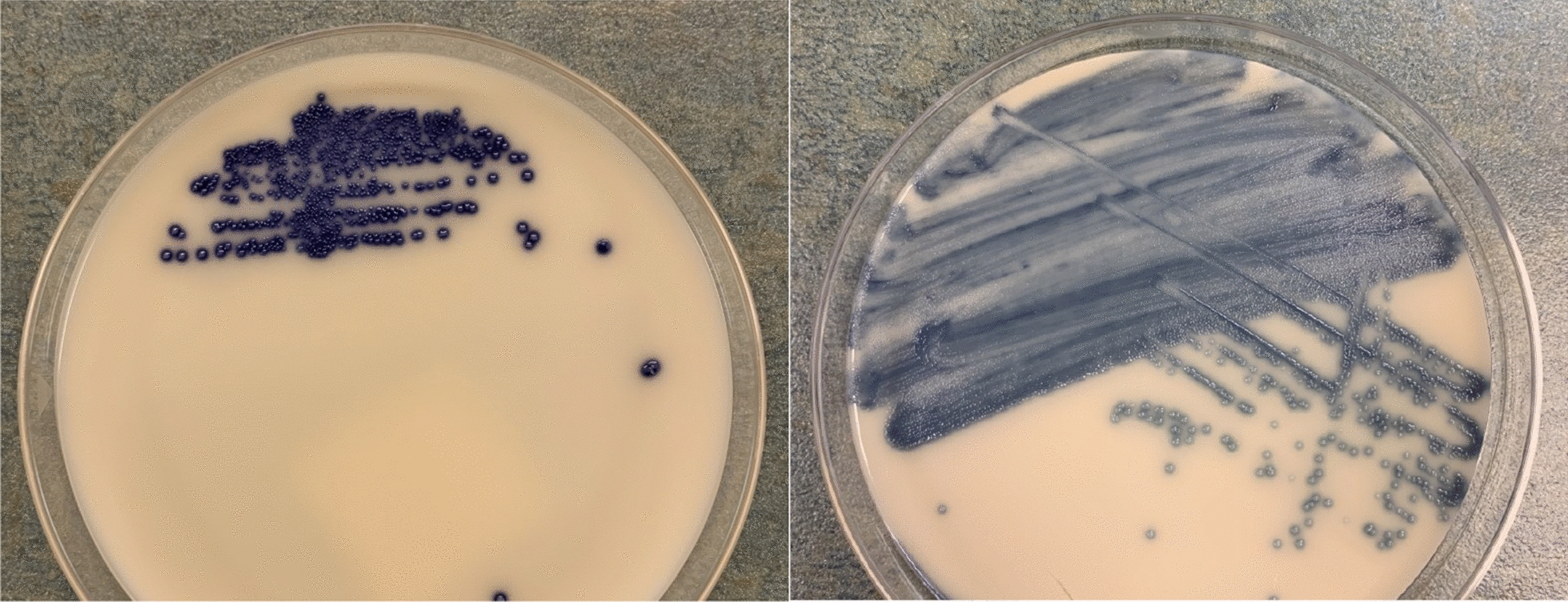
Images of Spectra™ VRE medium. Dark blue colonies are indicative of VRE *faecium* (left), and light blue colonies are indicative of VRE *faecalis* (right)

Spectra™ VRE agar (Remel, Lenexa, KS) is a selective and differential opaque chromogenic medium developed to recover and detect VRE from clinical specimens (rectal swabs and stool) for the purpose of screening for rectal colonization and infection control [9]. The medium contains 6 µg/mL of vancomycin and other antibiotics to inhibit growth of normal gastrointestinal flora [9]. Phosphatase enzymes in both *E. faecium* and *E. faecalis* interact with chromogens present in the media resulting in a light blue colored colony, but *E. faecium* also possesses a beta-galactosidase which results in a navy blue or pink-purple colony [9] (Fig. 1). Our laboratory sought to develop a workflow to optimize the use of the medium.

## Materials and methods

### Workflow optimization

**Fig. 2:**
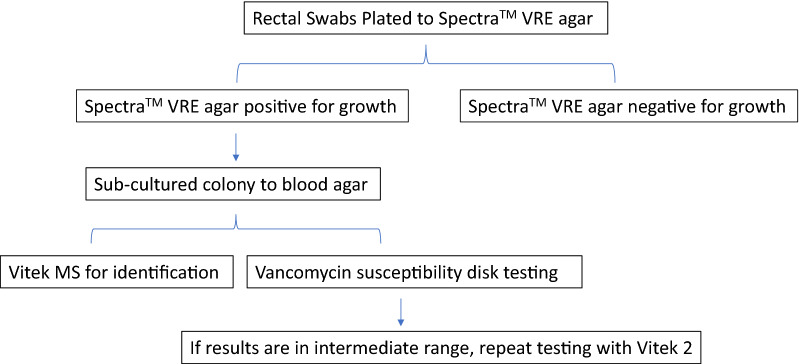
Workflow of detecting VRE in rectal swabs with Spectra™ VRE agar

Rectal swabs were collected from patients over a 3-month period at our institution for infection control purposes at Northwestern Memorial Hospital. All chromogenic colonies underwent VITEK MS (bioMérieux, l'Etoile, France), an automated mass spectrometry microbial identification system that uses matrix-assisted laser desorption ionization time of flight mass spectrometry (MALDI-TOF), for species identification. Kirby-Bauer disk diffusion was used to determine vancomycin susceptibility (Fig. 2). Clinical and Laboratory Standards Institute (CLSI) guidelines were followed for determining enterococcal vancomycin susceptibility by disk diffusion (zone diameter ≤ 14 mm resistant, 15–16 mm intermediate, and ≥ 17 mm susceptible) [10]. If vancomycin susceptibility was in the intermediate range, the culture was subjected to VITEK^®^2 (bioMerieux, l'Etoile, France) antimicrobial susceptibility testing and CLSI guidelines followed for vancomycin interpretation (≥ 32 µg/ml resistant, 8–16 µg/ml intermediate, and ≤ 4 µg/ml susceptible) [10].

A “confirmed culture” was defined as both the medium accurately identifying the species as either *E. faecalis* or *E. faecium* based on colony color (light blue for *E. faecalis* and dark blue, indigo, or purple for *E. faecium*) confirmed with VITEK MS and the medium accurately identifying vancomycin-resistance confirmed with either disk diffusion or VITEK®2. “Non-confirmed cultures” included the isolates that were not vancomycin resistant and/or were incorrectly identified with color-based speciation. Weekly quality control was performed during the study with *E. feacalis* ATCC® strains 51299 and 29212 and a VITEK MS confirmed *E. faecium* patient strain.

### Semi-quantitation of growth

The Spectra™ VRE medium manual claims a lower limit of detection of 50 colony forming units per milliliter [9]. The specimens were plated initially to the Spectra™ VRE medium in three quadrants (designated one, two, and three based on order of streaking). Growth was quantified as follows: less than five colonies only in quadrant one was called rare, greater than five colonies only in quadrant one was called few, growth in quadrants one and two was called moderate, and growth in quadrants one, two, and three was called many.

## Results

**Fig. 3:**
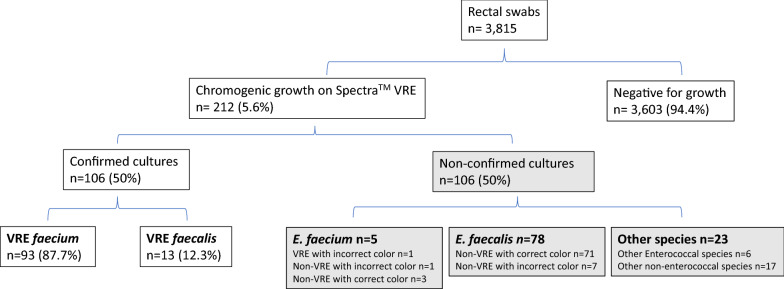
Detection of VRE with Spectra™ VRE agar in rectal swabs. Growth patterns affected accuracy of the agar. Confirmed cultures included those that correctly identified the species based on colony color and correctly predicted vancomycin resistance. Confirmation was done via Vitek MS and susceptibility testing. Non-confirmed cultures included the cultures that were vancomycin susceptible by susceptibility testing or incorrectly identified with color-based speciation

In total, 3,815 rectal swab samples were tested and 212 (5.6%) were positive for chromogenic growth on Spectra™ VRE agar (Fig. 3). Of the 212 cultures with growth, 106/212 (50%) were confirmed and 106/212 (50%) were non-confirmed. Of the confirmed cultures, 93/106 (87.7%) were *E. faecium* whereas 13/106 (12.3%) were *E. faecalis* (Fig. 3).

### Species identification via colony color on the Spectra™ VRE agar

Of the 212 cultures identified with Vitek MS, 195 (91.2%) were either *E. faecalis* (n = 91), *E. faecium* (n = 98), or other Enterococcus species (n = 6). Of the 195 cultures with Enterococci, colony color-based speciation was correct for 180 (92.3%) cultures while 15 cultures (7.7%) were misidentified. The 15 incorrect species included seven *E. faecalis* cultures with dark blue color colonies, two *E. faecium* cultures with light blue colonies, and six enterococcal species other than *E. faecalis* and *E. faecium* (five *E. raffinsonsus* and one *E. casseliflavus*, two of which were light blue and four were dark blue). The 17 cultures with non-enterococcal species (including twelve Staphylococcus species, two *Burkholderia cepacia* complex species, one Pediococcus species, one *Enterobacter cloacae*, and one Lactobacillus species) had light blue colonies in 15 (88.2%) and dark blue in 2 (11.8%) cultures. Overall, 96/109 (88.1%) of the cultures with colony color consistent with *E. faecium* were confirmed as *E. faecium*, and 84/103 (81.6%) of the cultures with colony color consistent with *E. faecalis* were confirmed as *E. faecalis*.

### Vancomycin resistance detection with the Spectra™ VRE agar

In total, 107/189 (56%) of *E. faecalis* (n = 91) or *E. faecium* (n = 98) cultures were confirmed to be vancomycin-resistant. The Spectra™ VRE agar showed differential selection for VRE *faecium* and VRE *faecalis*. While 94/98 (95.9%) of *E. faecium* isolates recovered by the medium were confirmed to be vancomycin-resistant, only 13/91 (16.5%) of the *E. faecalis* isolates were confirmed to be vancomycin-resistant (Fig. 3). Interestingly, most of the *E. faecalis* or *E. faecium* isolates incorrectly identified by color-based speciation were vancomycin susceptible. These included the seven *E. faecalis* cultures with dark blue colonies and one *E faecium* culture with a light blue colony. The data is also summarized in Table 1.

**Table 1:** Spectra™ VRE medium versus conventional methods

Spectra™ VRE medium vs. conventional methods				
	Accurate speciation	*E. faecalis* or *E. faecium*	Other species	Vancomycin resistant *E. faecium* or *E faecalis*
Dark blue/purple colonies	96/109 (88%)	103/109 (94%)	6/109 (6%)	93/103 (90%)
Light blue colonies	84/103 (82%)	86/103 (83%)	17/103 (17%)	14/86 (16%)

### Accuracy of the Spectra™ VRE agar depended on growth quantity

**Fig. 4:**
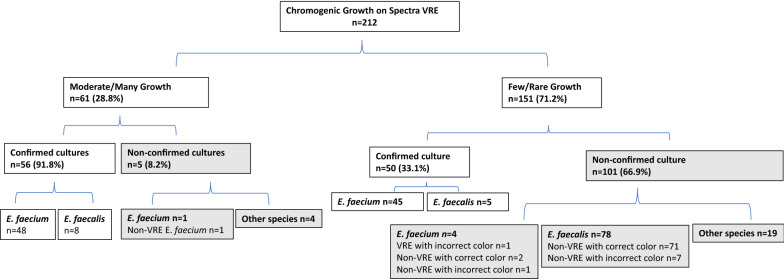
Growth patterns affected accuracy of the agar. Confirmed cultures included those that correctly identified the species based on colony color and correctly predicted vancomycin resistance. Confirmation was done via Vitek MS and susceptibility testing. Non-confirmed cultures included the cultures that were vancomycin susceptible by susceptibility testing or incorrectly identified with color-based speciation.

We found that the quantity of growth impacted the accuracy of the results of the agar. Growth in moderate to many amounts were detected in 61 (28.8%) cultures, 56 (91.8%) of which were confirmed (Fig. 4). Among the five non-confirmed cultures, one was vancomycin susceptible, and four grew species other than *E. faecalis* or *E. faecium*. In contrast, confirmation was achieved at a much lower rate among the cultures growing in few or rare amounts. Of the 151 (71.2%) cultures that grew in few or rare amounts, 50 (33.1%) were confirmed while 101 (66.9%) were non-confirmed (Fig. 4). Interestingly, 45 of the 50 confirmed cultures in this group were VRE *faecium*. *E. faecalis* was particularly affected by growth quantity as all eight isolates that grew in moderate to many amounts were confirmed, but only 5 of the 83 (6%) isolates growing in few to rare amounts were confirmed (Fig. 4).

### Impact of results on workflow

Overall, color-based speciation and vancomycin resistance were confirmed in 93 of 98 (94.9%) of *E. faecium* cultures recovered by Spectra™ VRE agar in any quantity, whereas only 13 of 91 (14%) of *E. faecalis* cultures were confirmed (8/13 had moderate to many amounts of growth). These data suggest the Spectra™ VRE agar has higher specificity for vancomycin resistance for *E. faecium* compared to *E. faecalis*.

**Fig. 5:**
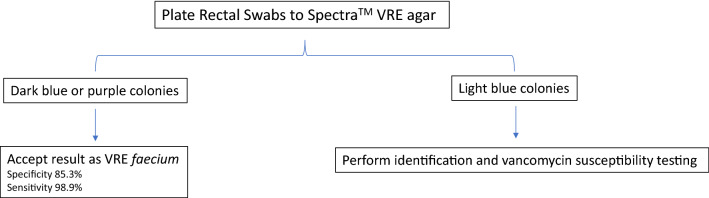
Proposed workflow using Spectra™ VRE agar to detect VRE in rectal swabs

Based on the results, light blue colonies recovered by the Spectra™ VRE agar require confirmation given the 82% specificity *E. faecalis* identification and the 16.5% specificity for VRE *faecalis*. Although 100% of *E. faecalis* isolates growing in moderate to many amounts were confirmed, the overall low number of isolates in that category preclude using growth quantity as a factor in the workflow until further data are available. Dark blue, indigo, or purple colonies can be taken as presumptive vancomycin-resistant *E. faecium* because those colonies have 88% specificity for *E. faecium* and the agar predicts vancomycin-resistance correctly in 96% of *E. faecium* isolates. The overall specificity for accepting all dark blue colonies as VRE *faecium* therefore is 93/109 (85.3%) (Fig. 5). Only one VRE *faecium* was light blue, suggesting a sensitivity of 93/94 (98.9%) for dark blue, purple, or indigo colony-based identification, but per our algorithm this outlier would be detected as we will do further testing on all light blue colonies (Fig. 5). The workflow proposed in Fig. 5 will reduce manual confirmation and reduce time to reporting VRE *E. faecium*. Because dark blue, indigo, or purple colonies constituted 109/212 isolates, we could potentially reduce manual confirmation in 51% of cultures.

## Discussion

Vancomycin-resistant enterococci (VRE) emerged in the 1980’s due to selection pressure from widespread antibiotic use and by now are a leading cause of nosocomial multi-drug-resistant infections. Current infection prevention surveillance practices to prevent nosocomial transmission utilizing rectal swab culture are time consuming and potentially lead to delayed implementation of isolation precautions, increasing the risk of VRE transmission among hospitalized patients [11]. Faster alternatives, including PCR methodologies and chromogenic agar, subsequently were developed to expedite the screening process.

Spectra™ VRE agar (Remel, Lenexa, KS) is a selective and differential opaque chromogenic medium developed to recover and detect the presence of VRE from clinical specimens (rectal swabs and stool) for the purpose of screening and infection prevention [9]. The package manual states that light-blue to blue colonies can be presumptively identified as *E. faecalis*, and navy-blue to pink-purple colonies as *E. faecium* [9]. The advantage of this medium is that it provides species identification and vancomycin susceptibility in one step. One disadvantage, according to published studies, is that the medium cannot completely inhibit the growth of vancomycin-susceptible enterococci. In 2021, Ping et al. studied 61 patient rectal swabs plated to Spectra™ [12]. When growth was observed, the isolate was sub-cultured to BHI broth and underwent confirmation of identification with PCR with primers targeted to D:alanine-D:alanine ligase (*ddl*) primers or to16S rRNA if *ddl* primers were negative [12]. Vancomycin resistance was confirmed using PCR primers for *vanA* and *vanB* followed by microbroth dilution [12]. In this study, 84% of *ddl*-confirmed *E. faecalis* recovered from the Spectra™ media were not VRE, while 94.5% of *ddl*-confirmed *E. faecium* were VRE [12]. These data suggest that Spectra™ VRE agar is more specific for *E. faecium* than for *E. faecalis*. Given this, the package insert for Spectra™ VRE agar recommends confirming isolates with identification and susceptibility testing [9]. However, confirming every isolate is time and resource intensive and delays time to result.

Our laboratory underwent evaluation of Spectra™ VRE agar by confirming positive chromogenic results with Vitek-MS identification and disk diffusion antimicrobial susceptibility testing. Vancomycin-resistance was correctly predicted in 96% of *E. faecium* isolates but only 16% of *E. faecalis* isolates. Colony color-based speciation was 88% specific for *E. faecium* but only 82% specific for *E. faecalis*. We found that growth quantity was important for accuracy of the agar and that poor growth was associated with higher rates of species and susceptibility discrepancy. Of the non-confirmed cultures, 101/106 (95.3%) grew in few to rare amounts.

One limitation of the study is that all rectal swabs were initially plated to the Spectra™ VRE agar. Therefore, all results were dependent on growth on this specialized medium and were not compared to other commercially available media.

PCR based assays are increasingly being utilized for VRE screening. The Spectra™ VRE agar has a cost advantage compared to commercially available PCR assays to detect VRE. When compared to the Cepheid GeneXpert® PCR *vanA* assay (Sunnyvale, California), the Spectra™ VRE agar saves $28.92 per test. Our laboratory receives approximately 18,000 VRE screening requests per year, which would equate to a saving of $520,000 per year. However, Spectra™ VRE agar has longer turn-around time of 24 h in comparison with 1–2 h for the GeneXpert^®^ *vanA* PCR [13].

## Conclusions

In our experience, the Spectra™ VRE agar performs well at predicting VRE *faecium*, but is unreliable for speciation and vancomycin-resistance for *E. faecalis.* Therefore, our results suggest our workflow can be streamlined when utilizing Spectra™ VRE agar (Fig. 5). Dark blue, indigo, or purple colonies can be trusted as VRE *faecium*, but light blue colonies in any growth pattern require further identification and antimicrobial susceptibility evaluation. This method has a sensitivity of 98.9% and specificity of 85.3% for detecting VRE *faecium*. It remains unclear as the why the media underperforms for *E. faecalis* and therefore we would recommend further research on this subject. We anticipate this workflow will significantly enhance throughput as it decreases the need to routinely subculture to other media for Vitek-MS and disk diffusion by 51%. Such practices could potentially preserve more reagents and media, decrease time to reporting results, and improve efficiency of laboratory staff. We found that higher growth quantity increased the accuracy of the agar, but further study is required to determine if this metric can be used to streamline the VRE screening workflow.

## Data Availability

The datasets used and/or analyzed during the current study are available on request from the corresponding author on reasonable request.

## References

[1] Cetinkaya Y, Falk P, Mayhall CG (2000). Vancomycin-resistant enterococci. Clin Microbiol Rev.

[2] O'Driscoll T, Crank CW (2015). Vancomycin-resistant enterococcal infections: epidemiology, clinical manifestations, and optimal management. Infect Drug Resist.

[3] Haque M, Sartelli M, McKimm J, Abu BM (2018). Health care-associated infections—an overview. Infect Drug Resist.

[4] CDC morbidity and mortality weekly report. August 6, 1993/42(30); 597–599. 8336690

[5] Diaz CA, Granados SM, Zimmer KM, Jernigan JA (2005). Comparison of mortality associated with vancomycin-resistant and vancomycin-susceptible *Enterococcal* bloodstream infections: a meta-analysis. Clin Infect Dis.

[6] Alevizakos M, Gaitanidis A, Nasioudis D, Tori K, Flokas ME, Mylonakis E (2017). Colonization with vancomycin-resistant enterococci and risk for bloodstream infection among patients with malignancy: a systematic review and meta-analysis. Open Forum Infect Dis.

[7] Weinstein RA, Diekema DJ, Edmond MB (2007). Look before you leap: active surveillance for multidrug-resistant organisms. Clin Infect Dis.

[8] Huang SS, Rifas-Shiman SL, Pottinger JM, Herwaldt LA, Zembower TR, Noskin GA, Cosgrove SE, Perl TM, Curtis AB, Tokars JL, Diekema DJ, Jernigan JA, Hinrichsen VL, Yokoe DS, Platt R (2007). Centers for disease control and prevention epicenters program, improving the assessment of vancomycin-resistant enterococci by routine screening. J Infect Dis.

[9] Spectra^TM^ VRE medium manual. Remel (Lenexa, Kansas).

[10] Clinical and laboratory standards institute (CLSI). performance standards for antimicrobial susceptibility testing. 31st ed. CLSI Supplement M100. 2021.

[11] D'Agata EM, Gautam S, Green WK, Tang YW (2002). High rate of false-negative results of the rectal swab culture method in detection of gastrointestinal colonization with vancomycin-resistant enterococci. Clin Infect Dis.

[12] Ping S, Mayorga-Reyes N, Price VJ (2021). Characterization of presumptive vancomycin-resistant enterococci recovered during infection control surveillance in Dallas, Texas, USA. Access Microbiol.

[13] Cepheid GeneXpert^®^*vanA* PCR manual (Sunnyvale, California).

